# Escherichia coli Has a Unique Transcriptional Program in Long-Term Stationary Phase Allowing Identification of Genes Important for Survival

**DOI:** 10.1128/mSystems.00364-20

**Published:** 2020-08-04

**Authors:** Karin E. Kram, Autumn L. Henderson, Steven E. Finkel

**Affiliations:** a Department of Biology, California State University, Dominguez Hills, Carson, California, USA; b Molecular and Computational Biology Section, Department of Biological Sciences, University of Southern California, Los Angeles, California, USA; University of Michigan—Ann Arbor

**Keywords:** cold shock, experimental evolution, genetics, long-term stationary phase, transcriptomics

## Abstract

Microbes live in complex and constantly changing environments, but it is difficult to replicate this in the laboratory. Escherichia coli has been used as a model organism in experimental evolution studies for years; specifically, we and others have used it to study evolution in complex environments by incubating the cells into long-term stationary phase (LTSP) in rich media. In LTSP, cells experience a variety of stresses and changing conditions. While we have hypothesized that this experimental system is more similar to natural environments than some other lab conditions, we do not yet know how cells respond to this environment biochemically or physiologically. In this study, we began to unravel the cells’ responses to this environment by characterizing the transcriptome of cells during LTSP. We found that cells in LTSP have a unique transcriptional program and that several genes are uniquely upregulated or downregulated in this phase. Further, we identified two genes, *cspB* and *cspI*, which are most highly expressed in LTSP, even though these genes are primarily known to respond to cold shock. By competing cells lacking these genes with wild-type cells, we show that these genes are also important for survival during LTSP. These data can help identify gene products that may play a role in survival in this complex environment and lead to identification of novel functions of proteins.

**IMPORTANCE** Experimental evolution studies have elucidated evolutionary processes, but usually in chemically well-defined and/or constant environments. Using complex environments is important to begin to understand how evolution may occur in natural environments, such as soils or within a host. However, characterizing the stresses that cells experience in these complex environments can be challenging. One way to approach this is by determining how cells biochemically acclimate to heterogenous environments. In this study, we began to characterize physiological changes by analyzing the transcriptome of cells in a dynamic complex environment. By characterizing the transcriptional profile of cells in long-term stationary phase, a heterogenous and stressful environment, we can begin to understand how cells physiologically and biochemically react to the laboratory environment, and how this compares to more-natural conditions.

## Abstract (precis)

Experimental evolution studies have elucidated evolutionary processes, but usually in chemically well-defined and/or constant environments. Using complex environments is important to begin to understand how evolution may occur in natural environments, such as soils or within a host. However, characterizing the stresses that cells experience in these complex environments can be challenging. One way to approach this is by determining how cells biochemically acclimate to heterogenous environments. In this study, we began to characterize physiological changes by analyzing the transcriptome of cells in a dynamic complex environment. By characterizing the transcriptional profile of cells in long-term stationary phase, a heterogenous and stressful environment, we can begin to understand how cells physiologically and biochemically react to the laboratory environment, and how this compares to more-natural conditions.

## INTRODUCTION

Experimental evolution studies of bacteria have revealed many insights into evolutionary processes (1–3). Many of these experiments have been performed in environments where only one factor is being experimentally manipulated, for instance, starvation for one nutrient (4), heat shock (5), antibiotic stress (6), etc. However, in natural environments, cells are likely experiencing multiple, as well as differing, stresses. We and others have previously used long-term batch culture experimental evolution to explore how evolutionary processes work in a heterogenous environment, where cells might experience some of the same environmental factors such as oxidative stress, anaerobic conditions, or low availability of certain nutrients (7–9). In these experiments, we allow cells to move through the entire life cycle of Escherichia coli in complex media, in order for cells to experience multiple types of stresses, which change throughout the incubation period.

During the E. coli life cycle in the laboratory, cells transition through the lag, log, and stationary phases and then into the death phase, where ∼99% of cells die, lyse, and release their cellular contents into the medium (10). This allows the remaining ∼1% of cells to enter long-term stationary phase (LTSP), where they can use the detritus of lysed cells as carbon and energy sources (10–12). The stresses of long-term stationary phase likely include high pH, high oxidative stress but low oxygen, and a lack of readily metabolized nutrients; however, none of these have been characterized completely (10, 13, 14). We previously showed that cells with mutations that may help cope with these stresses are selected for during this phase and that as populations continue further into LTSP, there is turnover of different mutant genotypes depending on the medium conditions at any given time (7, 9).

While we have hypothesized that this dynamism of genotypes indicates that different subpopulations of cells are growing and dying within LTSP cultures, we do not know if this phase consists of a collection of cells reflecting the four previous phases (lag, log, stationary, and/or death) or if LTSP consists of cells acting uniquely. We know that cells in lag, log, and stationary phases have unique transcriptional programs (15), but while expression changes due to genetic effects have been analyzed (16), to our knowledge, the transcriptional profile of LTSP in E. coli has not been studied. Elucidating the transcriptional profile of LTSP could help determine whether it is a phase in and of itself or whether it is more representative of an amalgam of cells experiencing the other phases.

In this study, we analyzed the transcriptome of E. coli throughout its laboratory life cycle, including death phase and LTSP. We have determined that the expression of a small set of genes is uniquely regulated during LTSP, indicating that this phase does have its own transcriptional program. Further, we hypothesize that the genes which are upregulated only in LTSP compared to other phases may be important for survival during this phase. We identify three genes in the cold shock protein family, *cspB*, *cspF*, and *cspI*, which are upregulated in LTSP compared to the other four phases. Loss of two of these genes (*cspB* and *cspI*) affects survival in LTSP during competition with wild-type cells. These results further indicate that (i) we may be able to use these types of data to characterize the biochemical and physiological responses to long-term stationary-phase stresses and (ii) we may be able to identify novel functions for proteins which have been characterized only in earlier phases of the life cycle. Understanding how cells respond biochemically to LTSP may allow us to determine how applicable standard laboratory conditions are as models to better understand natural environments.

## RESULTS

### The transcriptome of cells in LTSP can be distinguished from that of cells in other phases.

**FIG 1:**
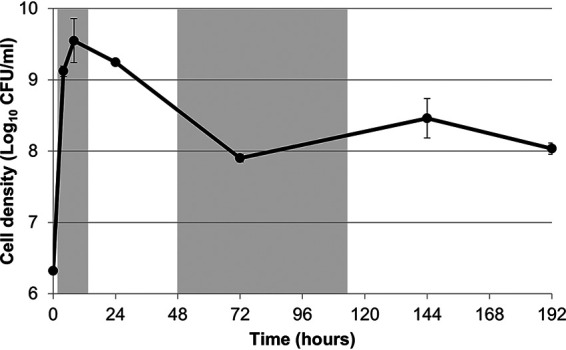
Average cell density during RNA-extraction time points. Three cultures were used for RNA extraction and for counting viable cells at 4, 8, 24, 72, 144, and 192 h postinoculation. Points represent averages of results from the three replicate cultures. Error bars represent standard deviations. In some cases, the error bar is so small that the point covers it. Shading represents a new phase in the E. coli life cycle (lag, log, stationary, death, long-term stationary).

In order to determine if there is a unique transcriptional program in LTSP, we incubated cells in triplicate into long-term stationary phase, extracted whole-cell RNA at six time points throughout the E. coli life cycle, and performed RNA sequencing (Fig. 1). Time points included log phase (4 h), late-log phase (8 h), stationary phase (24 h), death phase (72 h), and two time points in LTSP (144 and 192 h). We sequenced rRNA-depleted RNA from each cell population and analyzed at least 6 million reads per sample using HTSeq and DESeq2 (see Materials and Methods for a more detailed explanation) (17, 18).

**FIG 2:**
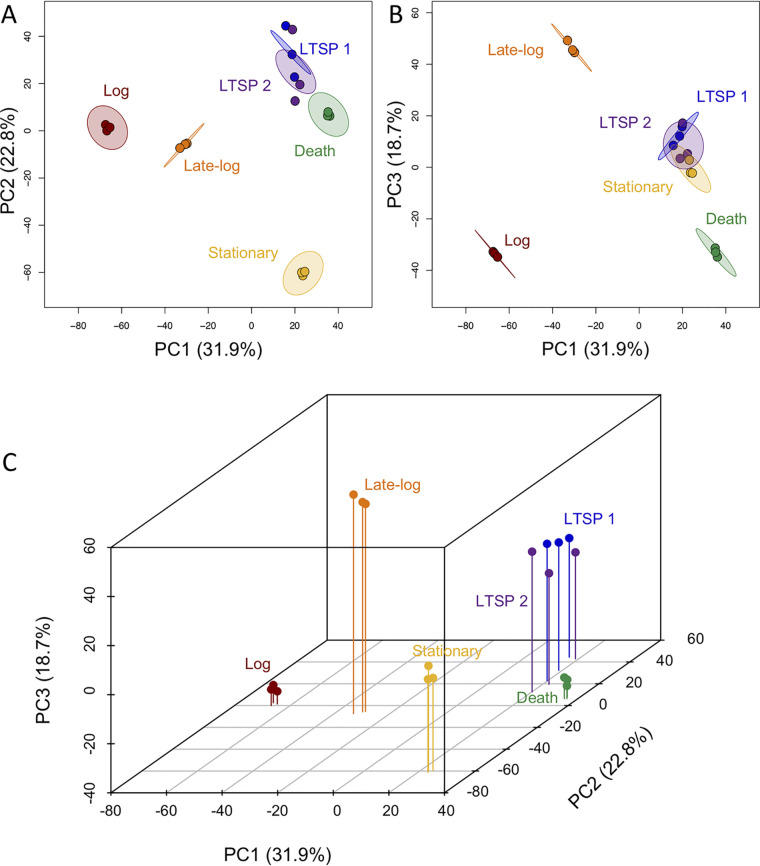
PCA of transcriptomes. Shaded circles represent 95% confidence intervals based on correlation matrices of the three replicates for each time point as follows: red, 4 h; orange, 8 h; yellow, 24 h; green, 72 h; blue, 144 h; purple, 192 h. (A) PC1 versus PC2. (B) PC1 versus PC3. (C) PC1, PC2, and PC3 plotted on the *x*, *z*, and *y* axes, respectively. The PCA results suggest that the cells had distinct gene expression patterns in each phase.

Using the gene expression data, we performed a principal-component analysis (PCA) to determine if there were features that distinguished the LTSP transcriptome from those of the other phases (Fig. 2). PCA ordinates the gene expression data across all 4,437 genes included in the analysis to identify principal-component axes (PCs) that capture the majority of the variance across all genes. We focused on the first 3 PCs as they capture 73.4% of the variation between samples (PC1 = 31.9%, PC2 = 22.8%, and PC3 = 18.7%). We can plot each sample across any combination of these axes to observe how similar each sample is to another sample with regard to the expression data and those principal components. Comparing PC1 to PC2 (Fig. 2A), we observed that cells in death phase and LTSP have similar positions along the two axes. However, cells in death phase were separated from cells in LTSP along PC3 (Fig. 2B), indicating that the expression patterns exhibited by cells in these two phases were similar with respect to PC1 and PC2 but different with respect to PC3. Cells in stationary phase were found to have a position similar to that seen with those in LTSP along PC1 and PC3 (Fig. 2B), indicating that there were similarities among their expression profiles for components 1 and 3 but that they were positioned uniquely along PC2 (Fig. 2A). Cells in log and late-log phase were positioned similarly along PC2 (as were cells in death phase; Fig. 2A) but were positioned uniquely along PC1 and PC3 (Fig. 2B). These data indicate that cells in the log and late-log phases had expression profiles that are similar to each other with respect to PC2 but different from each other with respect to PC1 and PC3. Interestingly, the expression profiles of cells in log and death phase were also similar along PC3 (Fig. 2B), indicating that there were similarities in expression patterns between these populations of cells with respect to this component. The fact that cells in LTSP had some gene expression patterns in common with those in stationary phase (PC1 and PC3) and in death phase (PC1 and PC2) supports the hypothesis that there are subpopulations of cells experiencing other phases during LTSP. However, while showing similarities in gene expression along some component axes, comparing each phase across all three axes simultaneously shows that each phase had a distinct expression profile (Fig. 2C).

We also plotted a 95% confidence interval (CI) ellipse for each time point using a correlation matrix representing our three replicates (Fig. 2A and B). By comparing overlaps among ellipses for different time points, we were then able to determine how similar the transcriptomes were across cells at different time points. Where two CIs overlap, this means that, with respect to the two plotted PCs, the cells in those populations have similar expression patterns. These data indicate that cells mostly have unique transcriptional profiles in each stage of their life cycle (they stay within their own CI, which does not overlap another population’s CI) but that there may be time points throughout the life cycle when cells in one phase may also have subsets of gene expression in common with cells in other phases.

We noted that the 3 replicates for each time point before the cells reached LTSP clustered within their 95% CI, indicating that biological replicates respond very similarly to their environment even as it changes. Further, we observed that populations in LTSP are not as similar to each other as those in other phases, as these replicates did not all appear within the 95% confidence interval. We also observed that the 95% CIs for each time point overlapped only in the following cases: each LTSP time point overlapped the other LTSP time point’s CI in each PC comparison (Fig. 2A and B), and the LTSP CIs overlapped stationary-phase time points in the comparison of PC1 to PC3 (Fig. 2B).

The PC analysis indicates that cells harvested at the two LTSP time points were very similar to each other. In order to determine how similar the expression profiles were at these time points, we identified the genes that were differentially expressed (DE) between cultures incubated for 144 and 192 h. Only 13 genes in 4 operons were determined to be significantly different (false-discovery-rate [*q*] value < 0.05; fold change > 2) between cells at these two time points (see Table S1 in the supplemental material). Since this is a relatively small number compared to differences during other phases (for instance, 567 genes were significantly DE between log-phase and h 144 cells), we treated the cells from the two LTSP time points as one group (LTSP) for the remainder of the analyses.

10.1128/mSystems.00364-20.1 TABLE S1 Genes differentially expressed between LTSP time points. Download Table S1, DOCX file, 0.01 MB. Copyright © 2020 Kram et al. 2020 Kram et al. This content is distributed under the terms of the Creative Commons Attribution 4.0 International license.

### Several genes are uniquely expressed in cells experiencing LTSP.

While the PC analysis indicates that the cell population in LTSP was transcriptionally distinct from those in the other phases, it was not clear whether there were genes uniquely expressed in this phase or whether this distinct profile was due to a mixture of cells in one of the other four stages.

**FIG 3:**
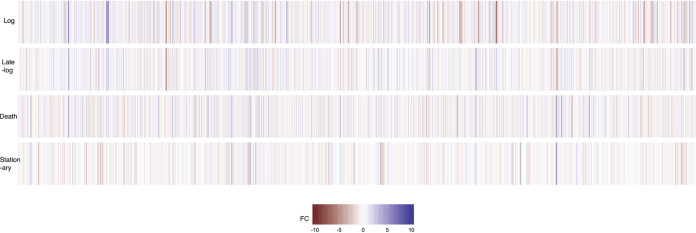
Heat map of the differences in expression between LTSP and other time points. Genes are indicated along the *x* axis in genome order. FC, log_2_ fold change.

In order to address this issue, we compared the expression levels for each gene between LTSP and each of the four other phases. All of the statistically significant DE genes are represented in Table S2. A heat map of each comparison representing all genes in the E. coli genome is shown in Fig. 3. Of genes DE in LTSP in comparison to log phase, 755 were found to be expressed at higher levels and 823 at lower levels during LTSP; of genes DE in LTSP in comparison to late-log phase, 669 were expressed at higher levels and 463 at lower levels in LTSP; of genes DE in LTSP in comparison to stationary phase, 632 were expressed at higher levels and 718 at lower levels in LTSP; and of genes DE in LTSP in comparison to death phase, 311 were expressed at higher levels and 499 at lower levels in LTSP. In each comparison, with the exception of late-log phase, more genes were expressed at lower levels in LTSP than were expressed at higher levels. Further, the number of genes expressed at higher levels than populations in LTSP decreased as populations approach LTSP.

10.1128/mSystems.00364-20.2 TABLE S2 Genes differentially expressed between LTSP and at least one other phase. Download Table S2, XLSX file, 0.1 MB. Copyright © 2020 Kram et al. 2020 Kram et al. This content is distributed under the terms of the Creative Commons Attribution 4.0 International license.

While some genes were consistently DE from LTSP, there are many sets of genes that were DE in only one or two other phases. These data support the hypothesis that the population of cells in LTSP likely consists of cells experiencing one of the other phases but that LTSP may also have a unique transcriptional program that cells in some subset are expressing.

In order to identify gene expression patterns unique to LTSP, we identified 38 genes that were significantly DE in LTSP compared to all other phases (Table 1); 32 of these genes were expressed at higher levels, and 6 of these genes were expressed at lower levels.

**TABLE 1:** Log_2_ fold change of genes differentially expressed in LTSP compared to all other phases

Regulation	Gene	Log_2_ fold change in indicated phase			
		Log	Late log	Stationary	Death
Up	*prpE*	6.86	4.99	5.80	1.76
	*efeU*	5.75	1.95	4.10	1.10
	*bdm*	5.06	3.31	1.75	2.12
	*glmY*	5.00	2.66	1.64	1.74
	*entD*	4.67	3.98	3.38	1.08
	*ydfK*	4.45	6.81	3.22	1.12
	*ynaE*	4.43	7.46	5.79	1.38
	*efeO*	4.13	1.24	3.58	1.47
	*alaE*	4.03	3.16	1.13	1.35
	*yaiY*	3.55	2.83	2.47	1.58
	*mltD*	2.95	1.77	2.29	1.45
	*ycfJ*	2.94	1.50	2.28	1.49
	*rpsT*	2.68	1.00	1.94	1.34
	*cspI*	2.61	5.82	3.76	2.01
	*ymgG*	2.56	2.75	2.92	1.31
	*efeB*	2.30	1.14	1.22	1.00
	*wzzB*	2.26	1.69	1.37	1.04
	*yhaL*	2.21	2.28	1.37	1.16
	*ynfN*	2.01	5.59	3.92	2.16
	*cspB*	2.01	6.69	4.97	2.24
	*gtrA*	1.88	1.93	2.12	1.77
	*queD*	1.64	2.11	2.72	1.48
	*yjbE*	1.57	1.27	1.52	1.77
	*ybgC*	1.56	1.57	1.28	1.35
	*leuZ*	1.43	1.91	1.03	1.34
	*valU*	1.40	1.79	1.28	2.15
	*valW*	1.30	3.93	2.76	1.45
	*metV*	1.28	1.12	1.92	1.11
	*ygbE*	1.23	1.89	1.82	1.44
	*cspF*	1.22	3.42	1.75	1.02
	*mliC*	1.10	1.27	1.49	1.32
	*ttcC*	1.02	2.42	1.07	1.02

Down	*narK*	−3.17	−2.18	−1.61	−2.15
	*yadK*	−2.36	−1.50	−2.81	−2.25
	*iraD*	−1.93	−1.65	−1.14	−1.13
	*ymgF*	−1.37	−1.04	−2.65	−1.25
	*caiB*	−1.37	−1.54	−1.09	−1.76
	*pfo*	−1.00	−1.56	−1.41	−2.11

### Some genes uniquely expressed during LTSP affect survival during this phase.

**FIG 4:**
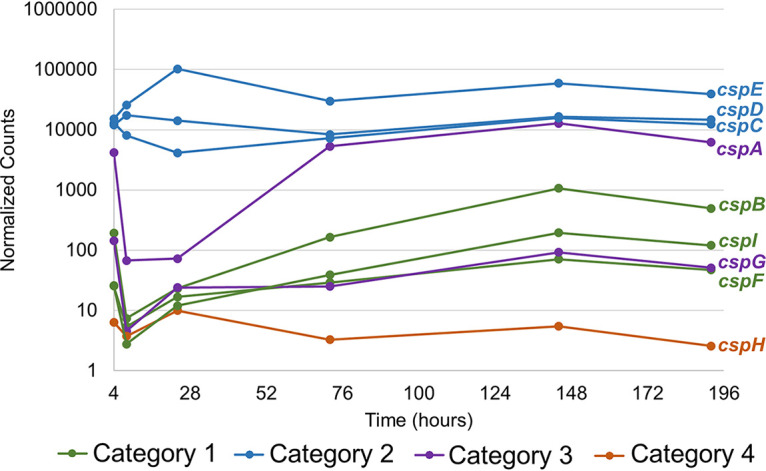
Expression pattern of *csp* genes. Normalized counts were outputted from DESeq2 analysis. Each point represents averages of results from the three cultures. We hypothesized that genes in category 1 would be important for survival in LTSP, genes in categories 2 and 3 might play a role in other phases of the life cycle, and the gene in category 4 would have no effect on cell growth or survival.

We hypothesize that genes which are expressed most highly in LTSP compared to other time points may play a role in cell survival. Interestingly, 3 genes identified as expressed at higher levels in LTSP than in all other phases were *csp* genes: *cspB*, *cspF*, and *cspI* (Table 1). *csp* genes were initially characterized as important for physiological adaptation of cells to cold temperatures (19), which cells in our cultures did not experience. Therefore, we were especially interested in this set of genes as they may be playing a role other than in response to cold shock in long-term batch cultures. The expression patterns of all 9 known *csp* genes during the incubation period are shown in Fig. 4. We grouped these genes into categories I to IV. Category I included the genes mentioned above which were expressed at higher levels during LTSP than during all other phases (*cspB*, *cspF*, and *cspI*). Category II included the genes which were highly expressed (normalized count > 1,000) at all time points (*cspC*, *cspD*, and *cspE*). Category III included the genes whose expression changed throughout the time course but whose expression in LTSP was not different from that in all other phases (*cspA* and *cspG*). Category IV included the one gene which showed low expression (normalized count < 15) at all time points (*cspH*). While the levels of *cspF* and *cspG* expression were similar in LTSP, *cspF* expression was lower earlier in the life cycle, which is why it is statistically placed in category I, whereas *cspG* expression started higher earlier in the life cycle, which is why it is statistically grouped in category III (Fig. 4). Each *csp* gene was expressed from its own operon—none shared promoters, although some might share regulatory regions—and are therefore likely regulated independently of one another.

**FIG 5:**
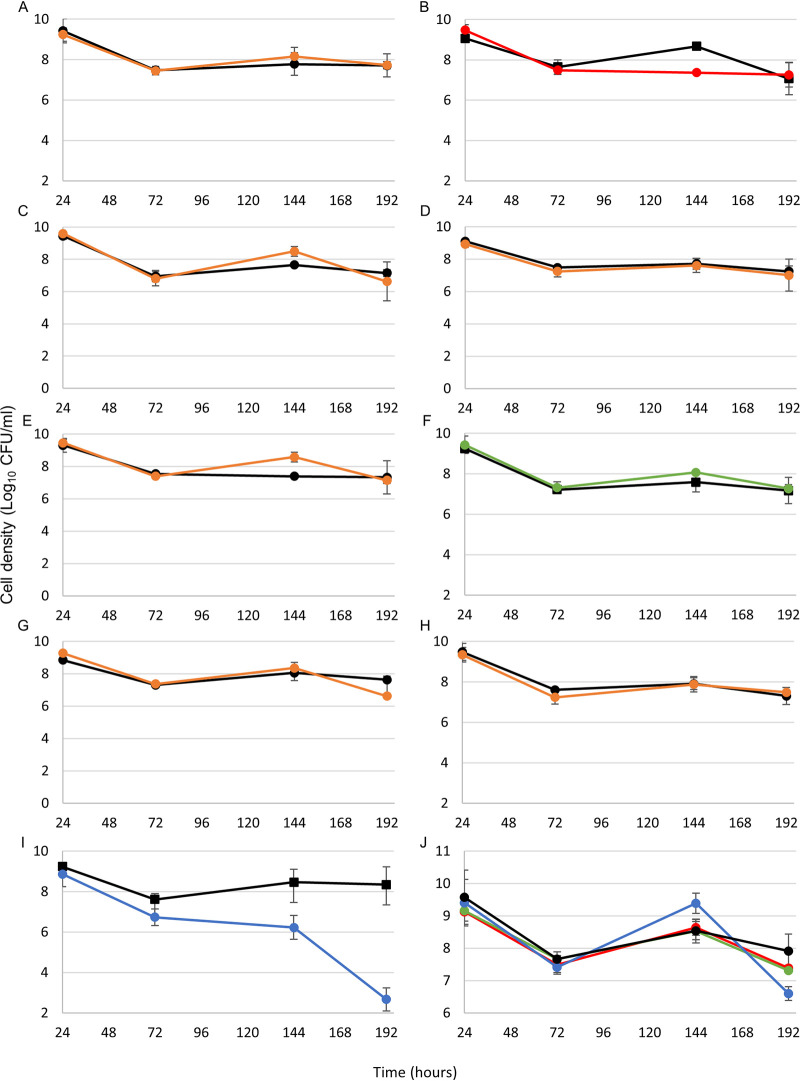
Competitions (A to I) and monocultures (J) of each *csp* mutant versus wild-type cells. Wild-type cells are shown in black in each graph. Letters correspond to the *csp* gene that is missing in that mutant cell (i.e., panel A shows the competition between the wild-type and *cspA*::Kan^r^ strains), with the exception of panel J, which shows monocultures of the *cspB*::Kan^r^ (red), *cspF*::Kan^r^ (green), and *cspI*::Kan^r^ (blue) mutants and the WT strain (black). Note that panel J has a different *y* axis scale. For panels B, F, I, and J, the points represent averages of results from three cultures. For A, C, D, E, G, and H, the points represent averages of results from two cultures. Error bars represent standard deviations. In some cases, the error bar is so small that the point covers it.

We predicted that all genes in category I would be important for survival in LTSP specifically, that genes in categories II and III may be important for growth or survival during other phases, and that genes in category IV would not affect cells under these culture conditions. To test these hypotheses, we deleted each gene from the PFM2 parental strain using P1 transduction from the Keio collection of gene knockouts (20) and incubated these cells into LTSP both alone in monoculture and in competition with wild-type cells. The data show that loss of genes in categories II, III, and IV had a negligible effect, if any, on growth of E. coli in competition with wild-type cells in long-term cultures (Fig. 5). Loss of *cspC* or *cspE* (both category II) might have given cells a slight (∼5-fold to 10-fold) advantage in early LTSP, but the advantage was gone 48 h later. Loss of the *cspF* category 1 gene also had no effect (Fig. 5F).

However, loss of two of the other category I genes, *cspB* and *cspI*, did affect the cell’s ability to compete with the wild-type strain, to various degrees (Fig. 5B and I). Cells mutant for *cspB* grew similarly to wild-type cells and experienced a similar death phase but then did not recover to the same level as the wild-type cells at 144 h (∼80-fold lower CFU/ml). In this case, wild-type cell density decreased over the next 48 h, which brought them to the same level as the mutant cells (Fig. 5B). Cells lacking *cspI* again grew similarly to wild-type cells but then had a slightly more severe death phase (∼10-fold-lower CFU/ml at 72 h) and never recovered fully from death phase, leading to complete loss of viable mutant cells by 192 h (Fig. 5I). Interestingly, when cells missing each of these genes were incubated in monoculture, without having to compete with wild-type cells, they did not show any defects in survival during death phase or LTSP (Fig. 5J).

## DISCUSSION

We have determined that each phase in the E. coli life cycle has a unique transcriptional profile (Fig. 2). This has previously been observed for log phase and stationary phase, but to our knowledge this is the first indication that gene expression in death phase and LTSP is significantly different from gene expression in other phases. Further, we have also shown that there are genes which are differentially expressed in LTSP compared to all other phases, indicating that this phase has a unique transcriptional program and does not represent only a combination of cells in other phases (although the populations are almost certainly also made up of cells in the other four phases, as well as of those experiencing this unique transcriptional program).

At least two of the genes that are expressed at higher levels in LTSP than in other phases, *cspB* and *cspI*, are important for survival in LTSP in competition against wild-type cells (Fig. 4 and 5). CspB and CspI are both cold shock proteins, which were initially discovered as induced at low temperatures (19, 21, 22). Generally, Csp proteins help to recover translation levels of proteins by binding to and relaxing mRNA that becomes too structured to be translated appropriately in colder temperatures, although some Csp proteins, including CspB and CspI, may be chaperones for single-stranded DNA (ssDNA) as well (19, 23, 24). Because cultures in these experiments are consistently incubated at 37°C, it is likely that these proteins are actually performing previously unidentified functions. Interestingly, both *cspB* and *cspI* are expressed in stationary phase even without cold shock (21), further supporting the idea that these genes are likely being expressed in response to stresses other than cold shock.

Other data indicate that these particular genes, as well as *csp* genes in general, may respond to other types of stresses. For instance, neither of these genes responded to cold stress in a pathogenic strain of E. coli (25). Further, Brandi et al. have shown that CspA, the originally identified cold shock protein, can also be regulated by the global regulators Fis and H-NS, indicating that it likely responds to stresses other than cold shock (26). *cspC*, *cspD*, and *cspE* are regulated by growth arrest during diauxic shift, H_2_O_2_ stress, and the transition to stationary phase (15). CspC and CspE are important for virulence in *Salmonella*, again indicating that they likely respond to a signal other than cold shock (27). Future studies will elucidate the roles of these and other genes during LTSP.

It is unsurprising that not all of the LTSP-specific genes identified play a specific role in LTSP—a similar finding was noted with transcriptome data in long-term cultures of Rhodopseudomonas palustris (28). However, data presented here indicate that, in fact, we can use expression patterns in LTSP to identify at least some genes that are important for survival in this environment and, further, possibly determine the growth state of cells within particular environments.

Determining which genes are expressed or repressed in lower-nutrient environments, as well as which proteins are essential for survival, will allow us to characterize the biochemical and physiological responses to stress in LTSP. We can then assess whether LTSP (or other laboratory conditions) may be a good model for natural environments, such as in a host or soil, by comparing those biochemical responses due to similar environmental factors, such as oxygen level and access to certain nutrients, to those in cells in natural environments.

## MATERIALS AND METHODS

### Bacterial strains, growth conditions, and viable cell counts.

All strains in this study originated from strain PFM2, derived from the E. coli K-12 lineage strain, MG1655 (29) kanamycin-resistant (Kan^r^) and chloramphenicol-resistant (Cam^r^) derivatives, or either mutant or “wild-type” cells, as appropriate. Before any experiment was initiated, overnight cultures were inoculated from frozen stocks into 5 ml of Luria-Bertani (Lennox) medium (LB) (Difco) in 18-mm-by-150-mm borosilicate test tubes, which were incubated with aeration in a TC-7 rolling drum (New Brunswick Scientific, Edison, NJ) at 37°C. We monitored cell growth and survival as described previously (13). Briefly, after inoculation of cells at a 1:1,000 (vol/vol) dilution from overnight growth frozen stocks into 5 ml of LB, we determined viable cell counts by serially plating dilutions of cultures on LB agar plates supplemented with kanamycin (50 μg/ml) or chloramphenicol (50 μg/ml), as appropriate.

### RNA preparation and sequencing.

We inoculated all cultures for transcriptome analysis from a single PFM2 overnight culture. At each time point, we sacrificed 3 cultures to remove 1 ml (4 h), 0.5 ml (8 h), or 2 ml (24, 72, 144, and 196 h) of cells, and viable cell counts were determined by serial dilution as described above. RNAprotect reagent (Qiagen) was added per the manufacturer’s instructions. Cells were pelleted and frozen at −80°C until total cellular RNA extraction was performed using an RNeasy Protect Bacteria minikit (Qiagen). Total RNA was processed, assessed for quality, and sequenced by the University of Southern California (USC) Genomics Core, where rRNA was removed using a Ribo-Zero kit (Illumina, Inc.). Single-end reads (75 bp) were generated from each sample on an Illumina HiSeq 2500 platform. We received an average of 7,882,901 reads per sample, with a range of ∼6.5 to ∼9.5 million reads per sample.

### Transcriptome analysis.

Using a custom Python pipeline, we used TopHat2 to align the raw reads to the E. coli MG1655 genome (30), SAMtools to generate binary alignment files (BAM) (31), and HTSeq to calculate read counts per gene and per sample (18). An average of 89.4% of reads across all samples mapped to our reference genome (Escherichia coli K-12 MG1655) (32), with a range of ∼66% to ∼96%. All rRNA gene reads, as well as reads of any genes that showed no expression (sum of gene counts in all samples < 1), were removed from the data set. We used the HTSeq output files as the input to analyze gene expression levels using DESeq2 (17), with comparisons between samples made based on the time of incubation. Once we had determined that expression differences between h 144 and h 192 were minimal, we marked these data points as the same time (“LTSP”) in our data frame. We identified differentially expressed genes using pairwise comparisons between time points. We considered genes to be differentially expressed between cells in LTSP and cells at other time points if the log_2_ fold change value was greater than or equal to 1 and if the *q* value was less than or equal to 0.05. We summarized the data using principal-component analysis (PCA) and regularized log scale (rlog) values of the normalized counts of each gene (derived from DESeq2) using the prcomp function with centering and scaling in base R. We added 95% confidence intervals by calculating correlation matrices for each the samples in each time point and then adding these intervals to our plot using the polygon function in the ellipse package in R (33). The three-dimensional (3D) PCA plot was created using the same values from prcomp, and the values were plotted using the Scatterplot3D package in R (34). We also created a heat map comparing the log_2_ fold change between the average normalized counts for each gene in LTSP versus each other time point using ggplot2 (35).

### Mutagenesis of *csp* genes.

We constructed in-frame knockout mutations of each *csp* gene individually (*cspA* to *cspI*) to create nine mutant strains by the use of P1 transduction from strains in the Keio collection (20). Briefly, we inoculated overnight cultures from a frozen stock in the Keio collection. The following day, we inoculated cultures at a 1:100 dilution into fresh medium and allowed them to grow to mid-log phase. Lysates were made from the donor cells using P1 stock. We then used the lysates to transduce wild-type PFM2 and selected for mutants on LB agar plates supplemented with kanamycin. We confirmed gene replacement using PCR.

### Competitions and monocultures with wild-type and mutant strains.

For monocultures, we inoculated cultures as described above. We also performed competitions between mutant strains and parental strains. Parental strains were marked with a chloramphenicol cassette replacing *lacZ* (*lacZ*::Cam^r^) (7), and mutant strains were marked with a kanamycin cassette replacing the gene of interest (*cspA*::Kan^r^ to *cspI*::Kan^r^). We inoculated wild-type cells (*lacZ*::Cam^r^) and cells of each of the mutants (csp*::Kan^r^) from frozen stocks as described above, and overnight cultures were each coinoculated at 1:1,000 (vol/vol) into LB broth in test tubes. We assayed both monoculture and competition populations for viable counts throughout the experiment (0, 4, 8, 24, 72, 144, and 192 h after inoculation) as described above.

### Data availability.

The data discussed in this publication have been deposited in NCBI’s Gene Expression Omnibus (36) and are accessible through GEO series accession number GSE152619.
