# Supplementary material for: Escherichia coli Has a Unique Transcriptional Program in Long-Term Stationary Phase Allowing Identification of Genes Important for Survival
Source: mSystems. 2020 Aug 4;5(4):e00364-20. doi: 10.1128/mSystems.00364-20 (PMC7406224; doi:10.1128/mSystems.00364-20)
Supplement: TABLE S1 [file mSystems.00364-20-st001.docx]

| Gene | Log2 FC* 144H v 192H | q value |
| --- | --- | --- |
| *carA* | 2.09 | 5.86E-14 |
| *carB* | 2.02 | 8.47E-14 |
| *paaB* | -3.94 | 9.18E-05 |
| *paaC* | -3.95 | 3.18E-05 |
| *paaD* | -3.35 | 1.87E-04 |
| *paaE* | -3.10 | 7.52E-04 |
| *paaF* | -2.89 | 1.46E-03 |
| *paaG* | -2.84 | 4.28E-03 |
| *paaI* | -2.65 | 1.75E-03 |
| *putA* | 2.35 | 2.93E-13 |
| *pyrB* | 3.44 | 1.53E-29 |
| *pyrE* | 2.00 | 5.04E-23 |
| *pyrI* | 2.36 | 7.15E-15 |
| *FC = fold change | | |
